# The impact of simulated MRI scanner background noise on visual attention processes as measured by the EEG

**DOI:** 10.1038/srep28371

**Published:** 2016-06-21

**Authors:** S. Oliver Kobald, Stephan Getzmann, Christian Beste, Edmund Wascher

**Affiliations:** 1 Leibniz Research Centre for Working Environment and Human Factors, Dortmund, Germany; 2 Cognitive Neurophysiology, Department of Child and Adolescent Psychiatry, Faculty of Medicine of the TU Dresden, Germany

## Abstract

Environmental noise is known to affect personal well-being as well as cognitive processes. Besides daily life, environmental noise can also occur in experimental research settings, e.g. when being in a magnetic resonance scanner. Scanner background noise (SBN) might pose serious confounds for experimental findings, even when non-auditory settings are examined. In the current experiment we tested if SBN alters bottom-up and top-down related processes of selective visual attention mechanisms. Participants completed two blocks of a visual change detection task, one block in silence and one block under SBN exposure. SBN was found to decrease accuracy in measures of visual attention. This effect was modulated by the temporal occurrence of SBN. When SBN was encountered in the first block, it prevented a significant improvement of accuracy in the second block. When SBN appeared in the second block, it significantly decreased accuracy. Neurophysiological findings showed a strong frontal positivity shift only when SBN was present in the first block, suggesting an inhibitory process to counteract the interfering SBN. Common correlates of both top-down and bottom-up processes of selective visual attention were not specifically affected by SBN exposure. Further research appears necessary to entirely rule out confounds of SBN in assessing visual attention.

We manage our daily life by continuously adjusting our behaviour to the environment. Incoming signals are evaluated with respect to their relevance for behaviour and draw attention relative to their saliency. According to the biased-competition model of selective visual attention^1^, attention is thought to be an interplay of two different pathways, 1) a bottom-up driven path dominated by stimulus saliency and 2) a top-down modulation to enhance the processing of task-relevant stimuli. In contrast to the automatic (stimulus-driven) bottom-up pathway of attention, top-down modulation is an intentional process that is modulated by cognitive effort applied to a given task or situation^2^. The attentional network is widespread across the brain, comprising sensory areas, the tempo-parietal junction and frontal regions such as the anterior cingulate cortex (ACC), and is thus susceptible to a variety of interfering factors^2^,^3^,^4^. These can improve or impair the selection of relevant information, which may affect processing^5^.

With increased extrinsic motivation (for example by rewards), frontal cognitive control mechanisms increase and the processing of relevant information is amplified^6^,^7^,^8^,^9^, leading to better performance in attentional selection. Loss of intrinsic motivation (for example with mental fatigue^10^) is rather restricted to a decay in higher order functions, i.e. cognitive control^11^,^12^. In addition to mental fatigue with its close link to motivation, acute stress, healthy aging, neurodegeneration, environmental noise or neuroactive solvents have also been shown to yield detrimental effects on attentional performance^13^,^14^,^15^,^16^,^17^,^18^, in a way that these weaken the top-down driven pathway of attention and force attentional shifts toward saliency-driven stimuli.

However, stress for example, is a very broad term. Usually, environmental noise is seen as a possible source of stress that is able to distract individuals from their current task by capturing attention, increasing arousal and interrupting different cognitive processes^17^,^19^,^20^,^21^. The actual effects of noise on visual tasks seem to depend on various features, such as the type and the temporal occurrence of noise. Sounds associated to visual stimuli may improve performance due to bottom-up driven multisensory integration in visual search for example^22^,^23^,^24^, while random noise (e.g. caused by traffic) can impair cognitive processes^25^,^26^. On the other hand, the mere interruption of processes might be buffered by individual cognitive capacities or task engagement^27^,^28^.

A thorough meta-analysis^21^ identified noise type, duration, intensity and schedule as important moderator variables for the impact of noise on behavioural performance. It has been shown that random noise is most distracting when it is presented discontinuously, since individuals cannot habituate to the random noise as easily as to continuous noise^21^. Habituation and hence adaptation to the detrimental effects of noise is more difficult when irrelevant noise changes its characteristics (e.g. pitch and amplitude), leading to larger interference and a stronger irrelevant sound effect due to attentional capture^29^,^30^.

Noisy conditions play a role in cognitive neuroscience research. Given that environmental noise is known to influence cognitive processing (and in particular visual attention^31^,^32^), it is surprising that the impact of scanner background noise (SBN) in (f)MRI research has not been adequately investigated. Previous research mainly focused on the influence of SBN on auditory processes and activations in auditory cortices^33^,^34^,^35^, and very little is known about the modulatory effects on visual attentional selection processes. Investigations of the impact of SBN on visual cortex activations revealed contradictory effects: Some studies reported no modulation of visual cortex activations due to SBN^36^, whereas others found decreased activations in visual cortex^37^,^38^ reported decreased response accuracy and increased regional cerebral blood flow in the ACC in the presence of simulated SBN in positron emission tomography (PET). In contrast^39^, found an improvement of behaviour in response to 70 dB of simulated SBN on cognitive control capacities. Thus, different sound pressure levels (SPLs) appear to result in contradictory effects in response to SBN exposure, with improved behaviour when being exposed to a rather low SPL and a decrease of behavioural performance in response to higher SPLs. According to a meta-analysis^21^, SBN, that can reach SPLs of up to 135 dB depending on magnetic field strength^34^, might disrupt attentional performance especially at the beginning of an experiment, since it takes some time to adapt to SBN. Even more so when the task is rather complex and connected to learning processes. However, previous investigations have not yet sufficiently distinguished specific effects of SBN on the different sub-processes of selective visual attention (i.e. bottom-up and top-down modulation), leaving it unclear, whether noise elicits a rather global effect or specifically influences the neurophysiology of top-down modulating cognitive control capacities or early stages of visual sensory processing. This is central to cognitive neuroscience research trying to examine the neuronal mechanisms underlying attentional selection.

Therefore, we applied a well-described visual change detection task in the present study to investigate the effect of SBN on the neurophysiology of cognitive processes of selective visual attention^40^,^41^,^42^. This task is able to distinguish between the particular sub-mechanisms of selective visual attention and has already been shown to be sensitive to influences of external factors like stress, aging and motivation^9^,^16^,^18^. In fMRI, the change detection task could elucidate differential neural activations related to individual levels of performance when resolving perceptual conflicts^43^. We chose an exposure to an average SPL of 80 dB as a middle ground between the assumingly challenging and improving SPL of 70 dB (cf.^39^) and higher SPLs in actual fMRI environments (at least 94 dB, although for example ear protection might decrease the subjective SPL; cf.^34^,^35^) and the aforementioned studies^37^,^38^ (both approximately 95–105 dB SPL on average). Participants need to detect a possible luminance change in a bilateral stimulus display while ignoring any orientation change. In conditions in which a luminance change is accompanied by a contralateral orientation change serving as a distractor, a perceptual conflict arises because of the strong saliency of the irrelevant orientation change. This conflict has to be resolved by recruiting pre-frontal cognitive control capacities in order to bias the luminance change (c.f.^1^,^44^). Perceptual bias is reflected in posterior asymmetries of the EEG (N1pc, N2pc^45^), whereas cognitive control affordances are mirrored in modulations of the fronto-central N2^46^.

We expected a detrimental influence of noise particularly on cognitive control functions due to a higher SPL of 80 dB compared to the approach of ref. 39. This effect should be more pronounced for participants starting with the noisy block, since adaptation and learning of the task are impaired in the first block with noise compared to a first block in silence. With respect to EEG parameters, a modulation of the fronto-central N2 is assumed to reflect altered requirements for cognitive control mechanisms under noise. To what degree the exposure to noise may alter sensory potentials to the task-relevant luminance change or to the irrelevant orientation change is still unclear. As the present task has been shown to reveal alterations of sensory potentials in response to various exogenous factors, it can be expected that attentional allocation might be perturbed by SBN, for example by an increased distractibility by task-irrelevant orientation changes or a non-sufficient perceptual bias toward the relevant luminance change.

## Results

### Behavioural data

**Figure 1:**
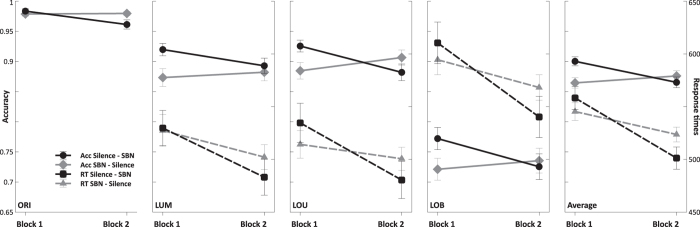
Performance data of the two presentation orders (silence–SBN and SBN–silence) across both blocks (Mean +/− SE). Accuracy data is represented by solid lines (Acc) and response times are represented by dashed lines (RT). Note that there are no correct response times in the ORI condition. The rightmost panel shows averaged accuracy and response times across all conditions.

Data of the two different presentation orders (silence–SBN and SBN–silence) relative to block and task condition are presented in Fig. 1. A significant interaction of block and order of SBN presentation was found for accuracy (*F*(1,22) = 5.26; *p* < 0.05; η^2^_p_ = 0.19). This interaction represents an overall influence of noise and shows that noise significantly decreased accuracy (with SBN: *M* = 0.86; *SE* = 0.01; without SBN: *M* = 0.89; *SE* = 0.01) There were no main effects of block (*F*(1,22) = 1.34; *p* = 0.26; η^2^_p_ = 0.06), order of SBN presentation (*F*(1, 22) < 1; *p* = 0.68; η^2^_p_ = 0.01) nor did the task condition influence the interaction of these factors (*F*(3, 66) < 1; *p* = 0.44; η^2^_p_ = 0.04). A Tukey post-hoc comparison revealed that the interaction of block and order of SBN presentation was caused by a significant decrease of accuracy only for participants being exposed to SBN in the second block (*p* < 0.01), while all other comparisons remained not significant (all *p* between 0.54 and 0.97). Thus, participants starting with silence were significantly less accurate in their second block under noise than in their first block in silence. On the other hand, participants starting with of block with SBN did not significantly improve their accuracy in the second block and more or less reached the level of accuracy of participants who started with a block of silence before (participants in silence in the second block: *M* = 0.88; *SE* = 0.01; participants under SBN in the second block: *M* = 0.87; *SE* = 0.01; comparison of the presentation orders in the second block: *p* = 0.89). However, as it was shown by the Tukey post-hoc comparison, accuracy did not differ in the first block (*p* = 0.54). Thus, starting in silence did not lead to significantly higher accuracy in the first block.

The analysis of response times did not show a significant interaction of block and order of SBN presentation (*F*(1, 22) = 2.76; *p* = 0.11.; η^2^_p_ = 0.11). A significant effect of block was found (*F*(1, 22) = 13.71; *p* < 0.01; η^2^_p_ = 0.38), showing that response times became faster in the second block of the experiment.

In summary, the presence of noise reduced the response accuracy in general, but had no significant effect on response times. The order of SBN presentation caused a significant decrease of response accuracy only for participants with SBN exposure in the second block. Response times became significantly faster in the second block in general, but were not significantly modulated by the order of SBN presentation.

### Noise Sensitivity Questionnaire (NOISEQ) results

**Figure 2:**
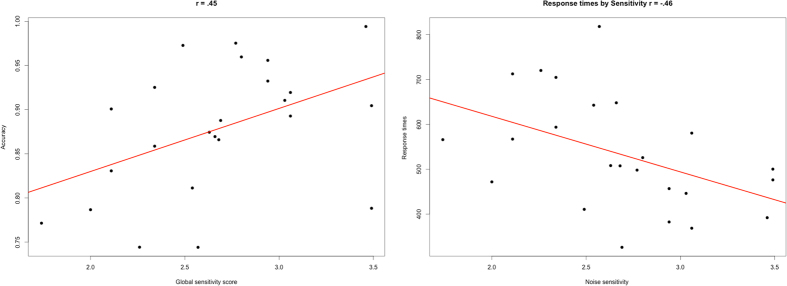
Scatterplots with regression lines of the NOISEQ results. On the left the correlation of accuracy with the noise sensitivity score is shown, whereas the correlation of response times and the noise sensitivity score is depicted on the right. Note that a higher global sensitivity score represents lower noise sensitivity.

We investigated the global sensitivity score of the NOISEQ^47^ to ensure that the participants of the different presentation orders did not differ (*F*(1, 22) < 1; *p* = 0.84; η^2^_p_ = 0.0). Lower noise sensitivity was positively correlated with accuracy (*r* = 0.45; *p* < 0.05) and negatively with response time (*r* = −0.46; *p* < 0.05), indicating that lower noise sensitivity was associated with increased response accuracy and decreased response times (Fig. 2).

### EEG results

#### ERL components N1pc and N2pc

**Figure 3:**
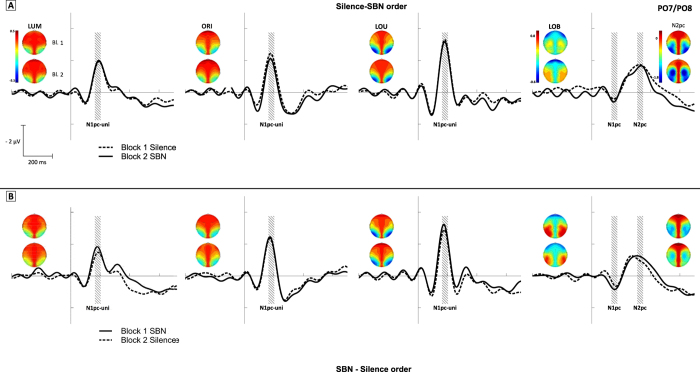
ERLs across all four task conditions are depicted separately for the two presentation orders (panel A: silence–SBN, panel B: SBN–silence). Note that dashed lines depict blocks in silence and solid lines blocks under SBN exposure. The grey areas depict the examined measurement windows. Topographies are given for the mean amplitude of the respective measurement windows in each task condition.

The ERLs of all conditions on electrode pair PO7/PO8 are depicted in Fig. 3, together with the topographies of the measurement windows. The amplitude of the N2pc as correlate of attentional re-allocation in the LOB condition of perceptual conflict was not significantly altered by the interaction of order of SBN presentation and block (*F*(1, 22) < 1; *p* = 0.62; η^2^_p_ = 0.01). The same was true for the amplitude of the N1pc as a correlate of the attentional capture of the irrelevant orientation change in the LOB condition (*F*(1, 22) = 1.21; *p* = 0.28; η^2^_p_ = 0.05). Also, neither block nor order of SBN presentation had a significant influence on the N1pc or N2pc amplitudes (all *F* < 1.93; all *p* > 0.18). Hence, characteristic modulations of EEG components related to perceptual conflict in the LOB condition are not modulated by the presence of SBN.

The N1pc-unilateral was analysed with the remaining three unilateral task conditions (i.e. LUM, ORI, LOU). It revealed only a significant main effect of block showing that the N1pc-unilateral decreased in the second block (*F*(1, 22) = 6.3; *p* < 0.05; η^2^_p_ = 0.22).

#### ERP components N2 and P3 on FCz and POz

**Figure 4:**
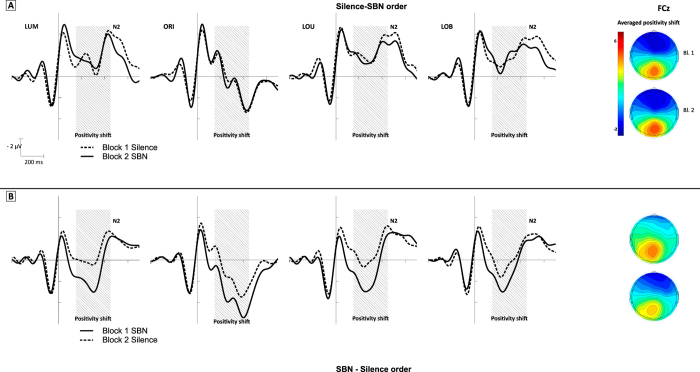
ERPs on FCz (Panel A: silence–SBN, panel B: SBN–silence). A pronounced positivity shift in the first block of the SBN-silence order can be detected. The grey area represents the measurement window of the positivity shift. On the right side the topography of the mean amplitude (averaged across task conditions) for the measurement window is given.

**Figure 5:**
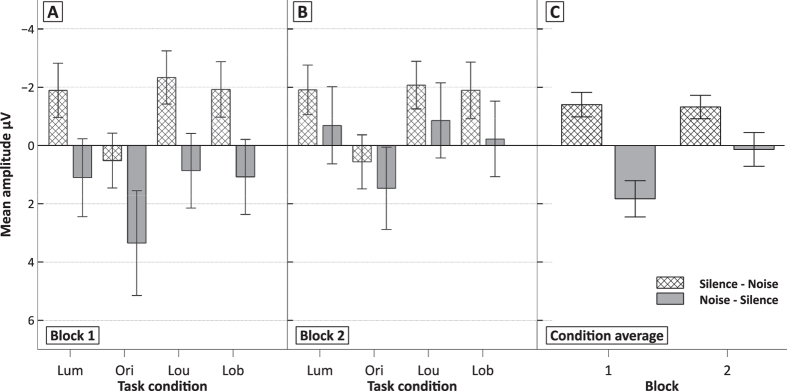
Mean amplitudes (+/−SE) of the positivity shift from 150–450 ms on FCz. Panel A shows data from block 1 and panel B from block 2. Panel C shows the cross-condition average in both blocks.

The ERP curves at the fronto-central electrode FCz are depicted in Fig. 4. On FCz, a comparatively large positive shift can be detected beginning after the change onset only in participants starting with SBN exposure. An ANOVA across a relatively large time window (150–450 ms after the stimulus change) that encompasses the initial processing of the stimulus change, before cognitive control is exerted by the following N2, resulted in an significant interaction of order of SBN presentation and block (*F*(1, 22) = 10.4; *p* < 0.01; η^2^_p_ = 0.32). This indicates that fronto-central activity was decreased when SBN was presented in the first block (Fig. 5). This was also supported by a Tukey post-hoc analysis with the factors order of SBN presentation and block, showing that the amplitude only differed significantly between the first and second block of the SBN–silence order (*p* < 0.01). The N2 on FCz and the P3 on POz as correlates of cognitive control did not show any significant modulation across all task conditions, blocks and presentation orders (all *p* > 0.11)

In summary, the ERPs and ERLs showed only mild and rather unspecific effects of SBN on neurophysiology. While sensory ERLs were unaffected by SBN exposure, fronto-central activity shifted to positivity when the first block was completed under SBN exposure, while such a shift did not occur in participants starting in silence. The N2 on FCz and the P3 on POz did not show a significant modulation by SBN exposure.

## Discussion

With the current experiment we tried to elucidate if SBN with a rather moderate SPL of 80 dB has an impact on attentional selection performance using a change detection task. The goal of this study was to examine which involved neurophysiological processes are most affected by SBN. Unlike other investigations of SBN we did not examine its effects in a scanner environment^36^,^37^, but applied the EEG in a highly controlled laboratory environment to profit from its high temporal resolution to examine selective visual attention.

Our results demonstrate that the presence of SBN mildly influences selective visual attention, albeit in an unspecific and inconsistent manner. As it was hypothesized and suggested by literature^21^, response accuracy suffered significantly from an exposure to SBN while response times were not significantly modulated by SBN exposure. At least on a descriptive level, a speed-accuracy trade-off might be implied, since accuracy was decreased while response times were accelerated. This could indicate a switch of strategy when encountering the aversive SBN, leading to faster response times while sacrificing higher accuracy at the same time. However, the temporal order of SBN exposure seems to also play a role for task performance. Regarding accuracy, participants of the different presentation orders showed a different pattern of performance in the first and second block, albeit only within and not between the experimental groups. Neither response times nor accuracy differed significantly between participants of the different presentation orders at any point of the experiment. Only on a purely descriptive level, participants starting in silence performed more accurately (across all task conditions) than participants starting under SBN exposure in the first block. At the same time, fronto-central activity of participants starting under SBN exposure was strongly shifted toward positivity. This effect could be referred to the potential aversiveness of SBN or to an increased arousal elicited by SBN^48^. Alternatively, this positivity shift could be a correlate of cortical inhibition of the task-irrelevant noise as a means of noise rejection and to shield against noise interference, which was also suggested by^17^,^49^ also showed that SBN suppresses activity in the default-mode network that is frontally located and is connected to a resting state compared to task-dependent activity^50^. The effect of the frontal positivity shift disappeared in the second block in the presence of silence while it does not appear at all in participants who started in silence. It could be assumed that task adaptation and task learning without interference of SBN in the first block did not result in the frontal positivity shift in the second block, because there was no need to suppress the task-irrelevant noise, while cortical inhibition was no longer needed for participants completing the second block in silence.

It could also be assumed that fatigue might have played a role in preventing or inhibiting this said frontal positivity shift in participants completing their second block under SBN exposure. Until now it remains unclear, if and how the current change detection task is modulated by fatigue per se across a longer period of time. In an investigation of the relationship of spontaneous eye-blinks and cognitive processing in experimental tasks, both accuracy and response times remained stable and unaltered across twice the amount of trials compared to the current implementation of the change detection task (cf.^51^). However, the comparison of these two task implementations appears difficult as stimulus presentation and the temporal protocol of the blocks differed in^51^. Also, there are no reported electrophysiological measures available for the change detection task in response to long-lasting periods of time that might induce fatigue. On the other hand, the general decrease of response times in the second block would contradict at least a behavioural effect of fatigue that usually leads to a slowing of response times^11^. Nevertheless, it cannot be conclusively ruled out that the lack of a positivity shift and the presumed cortical inhibition in the second block is caused by fatigue rather than unimpeded adaptation to the task in the first block. In order to elucidate if the assumed cortical inhibition are in fact prevented by an initially uninhibited adaptation, further experiments should examine the current change detection change during prolonged periods of time either without exposure to SBN or to several blocks of SBN.

Participants starting in silence performed significantly worse in their second block under SBN exposure compared to their first block, although they can adapt and learn the task without interference. This is contrary to our initial hypothesis, assuming that the effect of SBN should be more pronounced in participants starting under SBN exposure. This might be due to the fact that they still suffer from exposure to noise, especially since the possibly inhibiting positivity shift is absent in participants who are exposed to noise in the second block. In contrast, participants starting with SBN exposure elicited a more stable performance, with their performance not significantly changing from the first to the second block. Thus, it can be assumed that the temporal occurrence of an interfering factor such as SBN can manifest itself in different effects at least within individuals. Performance of participants starting with SBN exposure was more stable compared to the significantly decreased performance of participants facing SBN exposure in the second block. However, performance did not significantly improve after an exposure to SBN, either. In brief, SBN impaired accuracy when it was encountered in the second block, while it could prevent a significant improvement after an exposure in the first block of the experiment.

The exposure to SBN decreased accuracy across all task conditions in a similar manner. It can thus be assumed that SBN impaired attentional performance unspecifically without having differential effects on the bottom-up or top-down pathways of attention. If these mechanisms were selectively affected by SBN, we would have found specific behavioural and electrophysiological correlates. For example, accuracy in the most crucial LOB condition of perceptual conflict should have been more diminished in the presence of SBN if SBN would specifically affect top-down modulation. As it was already described in the introduction, this has been the case for stress or aging^16^,^18^. SBN exerted no such effect, which is also supported by the lack of significant modulations of key components of the EEG. Neither correlates of sensory processing (N1pc, N1pc-unilateral, N2pc), nor correlates of cognitive control (N2pc, N2, P3) differed between SBN exposure and silence. Hence, the effect of SBN might rather be explained in terms of increased arousal or coping strategies to reject noise which could have led to significantly decreased accuracy and descriptively decreased response times on a global level (i.e. without respect to the temporal order), with these processes being unrelated to cognitive control or sensory processing in a direct manner.

That being said, the occurrence of SBN per se in fMRI should not pose a serious threat to the cross-methodological interpretation of results regarding visual attention processes on the basis of the present results. However, there are still some issues that should be addressed in further examinations of fMRI noise. For example, the results of ref. 37 and ref. 38 both demonstrated significant influences of actual SBN in fMRI and of simulated SBN in PET on the visual cortex and ACC activity. However, we applied a quite moderate SPL of SBN of only 80 dB, in contrast to the SPLs used in the two aforementioned studies (range 95–105 dB) and SPLs in a real scanner environment (94–135 dB^34^). On the other hand, 80 dB could be a reasonable subjective SPL if ear protection is applied in fMRI (cf.^35^). Reference 39 could even demonstrate that 70 dB simulated SBN increased behavioural performance in three different tasks related to cognitive control capacities. The authors argue that the more challenging presence of noise might automatically facilitate recruitment of cognitive control. Thus, it remains to be elucidated if a SPL higher or lower than used in the present study might affect cognitive control or sensory processing mechanisms in terms of selective visual attention in the change detection task. Future experiments with different SPLs and different echo planar imaging sequences commonly used in fMRI other than the usually louder diffusion tensor imaging sequence used in this study could help estimating the actual effect of noise in real fMRI environments.

Our results support the notion that sensitivity to noise plays a role for potential effects of SBN. This was shown by the significant correlations of accuracy and response times with the sensitivity score given by the NOISEQ^47^. In brief, the lower the sensitivity the better the accuracy and the faster the response times. Lower sensitivity scores were positively correlated with accuracy, which acts against the indicated effect of SBN to lower accuracy and increase errors. In contrast, a low sensitivity also went along with faster response times, an effect that can be assumed to act in the same direction of SBN. Thus, individual sensitivity to noise in general and SBN in particular might be able to differentially and specifically skew results of studies in the scanner.

Another issue that cannot be entirely neglected by our simulation of SBN could inherently lie in the temporal occurrence of SBN, as the SBN presentation order had an effect on performance that should be considered when designing future experiments. Even with more or less “noise-reduced” approaches such as sparse temporal sampling (for a review of different approaches to reduce noise during experiments in fMRI see^35^), it cannot be ruled out at this point that SBN might skew results even on a smaller and a more accurate time-scale under certain conditions, e.g. as intermittent noise is known to be more disturbing than continuous noise^21^. Tasks requiring a lot of adaptation and practice might therefore be more vulnerable to the temporal occurrence of SBN than simpler tasks. This might also be true for tasks requiring internal speech (such as working memory tasks with verbal material) that have been shown to be more susceptible to the influence of SBN than the rather perceptually challenging change detection task^52^,^53^. Moreover, the individual noise sensitivity and potential experience with a given task might influence results in fMRI.

Additionally, with attention as an initial processing stage being contradictorily affected at lower SPLs, it is reasonable to assume that an influence of SBN on attentional processes also takes an effect in tasks, which rely on different cognitive capabilities like working memory or decision making. Thus, it cannot be precluded with certainty that results from a scanner environment over- or underestimate the effects of any actually investigated factor. Due to the fact that the present SPL was rather moderate in comparison to an actual scanner environment (especially without ear protection or with increasing magnetic field strengths applied in research), a systematic influence of SBN on both performance and the neuronal network of visual attention cannot be ruled out entirely. Varying SPLs and/or experimenting with different temporal sequences of SBN presentation orders (using a SBN–SBN sequence or a longer silence–SBN–silence sequence for example) could help to estimate the effects of SBN in future studies, especially in light of increasing magnetic field strengths which are accompanied by higher SPLs. A temporal sequence starting with a silent block for both groups and following alternations of SBN exposure would also allow for the assessment of a behavioural baseline. Such a baseline block could prove advantageous in answering the question whether differential pre-experience with a given task can modulate the effect of SBN exposure that led to a significant decrease of accuracy of participants starting with a silent block compared to their second block under SBN exposure. As the silent and noisy blocks were only intersected by a five-minute break in the present study, it appears advisable to take measurements of sessions on different days. Such an approach would resemble typical cross-methodological approaches more strongly (i.e. with EEG and fMRI measurements taking place on different days as well) and therefore increase the ecological validity of the results.

In summary, exposure to simulated SBN decreased accuracy in a task for measuring visual attention. This effect was modulated by the temporal occurrence of SBN. When SBN was encountered in the first block, it prevented a significant improvement of accuracy in the second block, whereas SBN significantly decreased accuracy when it appeared in the second block. Neurophysiological findings showed a strong frontal positivity shift only when SBN was present in the first block, suggesting an inhibitory process to counteract the interfering SBN, which might be prevented by an initial task adaption in silence. Common correlates of both top-down and bottom-up processes of selective visual attention were not specifically affected by SBN exposure. Therefore, fMRI examinations and findings appear uncritical in regard to proper attentional functioning at the time being. However, the present results demonstrate that more research with different SPLs and more temporal sequences of SBN occurrence with a higher ecological validity than in the present study might still be required to completely rule out influences of SBN on visual selective attention, which might ultimately hamper the cross-methodological interpretation of fMRI findings in the worst case.

## Methods

### Participants

24 participants (14 female, *Mean* = 24.7 years, *SD* = 3.01) were tested during this experiment. All participants were right-handed, had normal or corrected-to-normal vision, reported no hearing deficits and gave informed written consent. Before the experiment the participants filled in the NOISEQ^47^. After completing the experiment they either received course credit or payment of 10 € per hour. All experimental procedures were approved by the local ethic committee of the Leibniz Research Centre for Working Environment and Human Factors and were carried out in accordance with the Declaration of Helsinki.

### Procedure and stimuli

All experimental procedures were conducted at the Leibniz Research Centre for Working Environment and Human Factors and took approximately 150 minutes. After filling in some short questionnaires, the preparation of the EEG cap began and the participants received a written instruction of the change detection task. The participants completed two blocks of the change detection task intersected by a short break for approximately five minutes. A block was either completed with or without simulated SBN. The SBN was real MRI scanner sound that was recorded at the radiology lab of the University of Iowa Hospitals and Clinics (http://www.cornwarning.com/xfer/MRI-Sounds/DTI.mp3) by a microphone (Shure Beta 58A) placed inside the MRI room. The SBN consisted of a recording of Diffusion Tensor Imaging, chosen for its roughly uniform structure. As the original soundfile was no longer than 40 s, the file was duplicated to a constant duration of 15 min (with 10-ms on/off ramps to exclude clicks), using the software Cool Edit 2000 (Syntrillium Software Corporation, Phoenix, AZ, USA). The 15-min sound file was repeatedly presented in the noise block (with 300-ms on/off ramps at the beginning and the end of the file and a short silent period in-between). The sound was digitized at 48 kHz sampling rate and 16-bit resolution and was converted to analogue form via a PC-controlled soundcard (Sony Sound Reality Audio Enhancer, Sony, Minato, Japan). SBN was presented by over-ear headphones (AKG Acoustics GmbH, Vienna, Austria) that were worn by the participants throughout the whole experiment to keep the physical pressure on the EEG electrodes constant. The SPL varied from 75 to 90 dB, with an average level of 80 dB (measured by a sound level meter, Brüel & Kjær Sound & Vibration Measurement A/S, Nærum, Denmark). SBN was presented by a notebook outside the EEG cabin. To prevent an easy adaptation to SBN we chose a recording with varying pitches. The order of blocks with and without SBN was pseudo-randomized across participants. A block of the change detection task comprised 768 trials and lasted for approximately 40 minutes.

**Figure 6:**
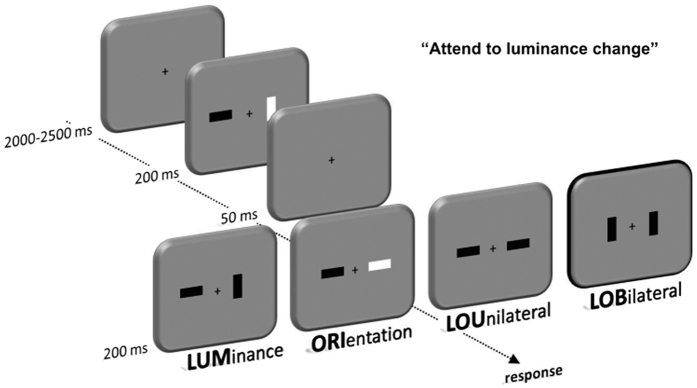
Depiction of the change detection task. Participants responded to the side of a luminance change. According to the type of change four different conditions arise: LUM, LOU, ORI and LOB.

A trial of the change detection task was composed of an array of two rapidly presented stimulus displays for 200 ms each and an intersecting blank screen for 50 ms. The inter-trial-interval (ITI) varied between 2000 and 2500 ms (Fig. 6). The participants were seated in front of a 22-inch CRT monitor with a refreshing rate of 100 Hz in a viewing distance of 120–140 cm. Stimulus presentation was controlled by a VSG 2/5 graphic accelerator (Cambridge Research Systems, Rochester, UK). A display consisted of two bars that are presented laterally relative to a fixation cross (visual angle 1.1°). The bars were either horizontally or vertically aligned and were presented with either high or low luminance (45 cd/m^2^ vs. 20 cd/m^2^, background 30 cd/m^2^).

The task of the participants was to indicate the side on which a luminance change occurred relative to the first stimulus display of a trial by button press. There were four change conditions: 1) one bar changes its luminance (luminance unilateral, LUM), 2) one bar changes its orientation only (no-go condition; orientation unilateral, ORI), 3) one bar changes both its luminance and its orientation (luminance – orientation unilateral, LOU), and 4) one bar changes its luminance, while the other bar changes its orientation (luminance–orientation bilateral, LOB).

### Behavioural data recording

Responses of the participants were recorded by force buttons affixed to the left and right side of the seat inside the EEG cabin. A button press of at least 150 cN was required for counting as a response. All button presses to the respective side of an occurring luminance change between 150 and 1500 ms after presentation were counted as “correct responses”, as were no button presses in the ORI condition. Missing responses (no button press or with latencies above 1500 ms), false alarms (button press in the ORI condition) or button presses to the opposite side of a luminance change were concatenated as “errors”.

### EEG data recording and analysis

EEG recordings were conducted by employing 60 active Ag/AgCl electrodes (Brain Products, Gilching, Germany). Attachment of the electrodes to the scalp took place according to the extended 10/20 system^54^. The horizontal and vertical electro-oculography was recorded by four electrodes (two electrodes affixed to the outer canthi of each eye and two electrodes above and below the right eye). The EEG was sampled online by a BrainAmp amplifier (Brain Products, Gilching, Germany) with a frequency of 1000 Hz and a low-pass filter of 200 Hz and an online common reference.

EEG data was re-referenced offline to averaged mastoids and filtered by applying a high-pass filter of 0.5 Hz and a low-pass filter of 8 Hz (cf.^9^,^40^). Event-related-potentials (ERPs) and event-related-lateralizations (ERLs^55^), time-locked to the stimulus change with a duration of 1250 ms (450 ms before the change and 800 ms after the change, baseline set between −450 and −250 ms, i.e. 200 ms preceding the first stimulus display) were computed and checked for artefacts. Eye movements and blinks were removed by applying a regression-based algorithm^56^. Only ERPs and ERLs of correct trials and their respective mean amplitudes were averaged across respective time windows and analysed using the EEGLAB^57^ and ERPLAB^58^ toolboxes for MATLAB (MathWorks, Natick, USA). The ERLs of the LOB condition were averaged across time windows from 135–175 ms (N1pc) and 310–350 ms (N2pc). The ERLs at electrode sites PO7/PO8 of the unilateral conditions LUM, ORI and LOU were averaged across a time window from 164–204 ms (N1pc-unilateral). The fronto-central N2 (time window: 400–500 ms) and a broad positivity shift (time window: 150–450 ms) were averaged at electrode site FCz, while the parieto-occipital P3 was averaged at electrode POz (time window: 353–393 ms).

### Design and statistical analysis

The experiment comprised three factors, the between-subjects factor “order of SBN presentation” (noise–silence vs. silence–noise) and the within-subjects factors “block” (Block 1 vs. Block 2) and “task condition” (LUM, ORI, LOU, LOB). Note that the interaction of order and block would represent a main effect of noise. Thus, a 2 × 2 × 4 mixed ANOVA for the rate of correct responses was computed, and a 2 × 2 × 3 mixed ANOVA for correct response times (given the fact that there were no correct response times in the ORI condition). The statistical analyses of the different mean amplitudes of ERPs and ERLs were adapted accordingly. Corresponding post-hoc analyses were computed subsequently where appropriate. All statistical analyses were carried out by RStudio (RStudio, Boston, USA) and considered significant at *p* values <0.05. *P* values were adjusted for multiple testing (by FDR method) and violations of sphericity (by Greenhouse-Geisser correction) where implied.

## Additional Information

**How to cite this article**: Kobald, S. O. *et al*. The impact of simulated MRI scanner background noise on visual attention processes as measured by the EEG. *Sci. Rep.* **6**, 28371; doi: 10.1038/srep28371 (2016).
